# ER/AR Multi-Conformational Docking Server: A Tool for Discovering and Studying Estrogen and Androgen Receptor Modulators

**DOI:** 10.3389/fphar.2022.800885

**Published:** 2022-01-24

**Authors:** Feng Wang, Shuai Hu, De-Qing Ma, Qiuye Li, Hong-Cheng Li, Jia-Yi Liang, Shan Chang, Ren Kong

**Affiliations:** ^1^ Changzhou University Huaide College, Taizhou, China; ^2^ Institute of Bioinformatics and Medical Engineering, School of Electrical and Information Engineering, School of Chemical and Environmental Engineering, Jiangsu University of Technology, Changzhou, China

**Keywords:** estrogen receptor (ER), androgen receptor (AR), molecular docking, similarity search, web-server

## Abstract

The prediction of the estrogen receptor (ER) and androgen receptor (AR) activity of a compound is quite important to avoid the environmental exposures of endocrine-disrupting chemicals. The Estrogen and Androgen Receptor Database (EARDB, http://eardb.schanglab.org.cn/) provides a unique collection of reported ERα, ERβ, or AR protein structures and known small molecule modulators. With the user-uploaded query molecules, molecular docking based on multi-conformations of a single target will be performed. Moreover, the 2D similarity search against known modulators is also provided. Molecules predicted with a low binding energy or high similarity to known ERα, ERβ, or AR modulators may be potential endocrine-disrupting chemicals or new modulators. The server provides a tool to predict the endocrine activity for compounds of interests, benefiting for the ER and AR drug design and endocrine-disrupting chemical identification.

## Introduction

With the development of chemistry technology, numerous natural or non-natural compounds are synthesized and used in the daily life of human beings such as medicines, perfumes, food additives, automobiles, electronics, pesticides, textiles, plastics, and so on (Barr Dana et al., 2005; Judson et al., 2011). It is noted that a number of the compounds act as endocrine-disrupting chemicals (EDCs) with the potential to interfere the hormone systems in human or wild lives (Schug et al., 2011; Dionisio et al., 2015). The occupational and environmental exposures of EDCs are strongly correlated with the adverse health outcomes such as reproductive health, development disorders, oncological, immunological and cardiovascular disease, obesity, and neurobehavior disorders (Elobeid and Allison 2008; Yilmaz et al., 2020; Boudalia et al., 2021; O'Shaughnessy et al., 2021; Priya et al., 2021). Numerous efforts have been taken to identify that if a compound is endocrine-active or not. The Endocrine Disruptor Screening Program (EDSP) and the Toxicology Testing in the 21st Century (Tox21) projects set up various *in vitro* or *in vivo* assays to measure the potential effects of chemicals on the endocrine system in humans or wildlife (Judson et al., 2010; Willett et al., 2011; Judson et al., 2015; Yilmaz et al., 2020). However, high costs and low speed make the experimental methods not fulfill the need of testing the rapid increased number of synthetic chemicals in use. Currently, only a small fraction of compounds have the experimental determined endocrine activity data available (Egeghy et al., 2012; Tickner et al., 2019). It is of great need to develop the predictive models to provide clues of the compounds’ endocrine activity.

The *in silico* methods, especially the ligand-based QSAR (quantitative structure–activity relationships) approaches and structural base docking methods, are widely used in the computer-aided drug design field (Mao et al., 2021; Sabe et al., 2021). These methods are applied to predict the compound’s activities against endocrine-related proteins, such as the estrogen receptor (ER) and androgen receptor (AR) (Schneider et al., 2019). The CERAPP (Collaborative Estrogen Receptor Activity Prediction Project) and CoMPARA (Collaborative Modeling Project for Androgen Receptor Activity) construct various predictive models trained by different QSAR approaches for estrogen or androgen receptor activity prediction (Mansouri et al., 2016; Mansouri et al., 2020). Both categorical and continuous models are built based on the dataset provided by the U.S. EPA, and a consensus model was obtained by weighting the models on scores based on evaluated accuracies of single models. Shen et al. collected the estrogenic activity data from public sources and developed QSAR models based on the dataset (Shen et al., 2013). Machine learning and deep learning methods are also applied in EDC predictions and achieved a relative high overall predictive accuracy (Zhang et al., 2017). One of the important limitations of the QSAR-based model is the quality of data for model training (Maggiora 2006). The structure-based docking method provides significant complementation for EDC prediction. The “Endocrine disruptome” server provides docking models for 14 nuclear hormone receptors such as ER, AR, glucocorticoid receptor, liver X receptors, etc. (Kolšek et al., 2014). However, only 18 structures are incorporated in the server.

**FIGURE 1:**
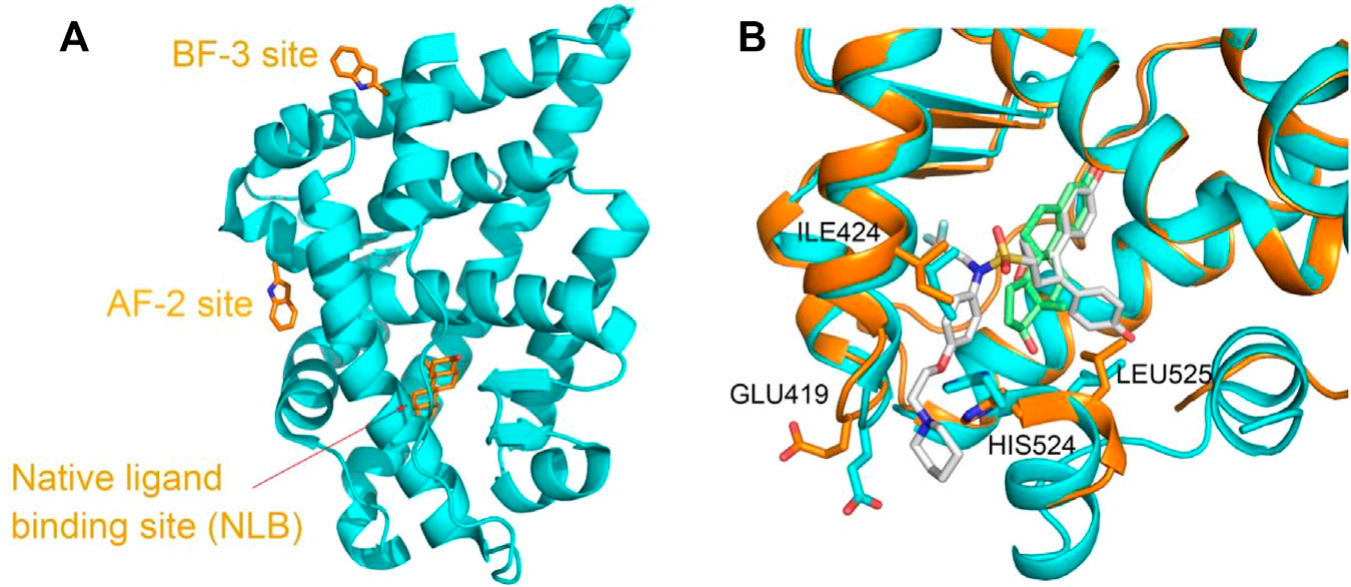
**(A)** Three binding sites in the ligand-binding domain of the AR depicted based on PDBID 2POI and **(B)** the ligand-binding site flexibility of ERα in complex with different compounds, estradiol ((8R,9S,13S,14S,17S)-13-methyl-6,7,8,9,11,12,14,15,16,17-decahydrocy clopenta[a]henanthrene-3,17-diol) and 7AI ((1S,2R,4S)-5,6-bis(4-hydroxyphenyl)-N-{4-[2- (piperidin-1-yl)ethoxy]phenyl}-N-(2,2,2-trifluoroethyl)-7-oxabicyclo[2.2.1]hept-5-ene-2-sulfonamide) (structures from PDBID 5GS4 and 7RRX). Proteins are represented in a cartoon model, and compounds or residues, in a stick model. In **(B)**, proteins from 5GS4 and 7RRX are colored in cyan and orange, and the compounds estradiol and 7AI are colored in green and gray, respectively. Residues undergoing considerable conformation changes, such as ILE 424, GLU419, HIS524, and LEU525 are depicted in the stick model.

ER and AR are also pivotal therapeutic targets due to the roles in regulation of development, endocrinology, and metabolism, and numerous compounds are developed to modulate the protein functions. The nuclear receptors including the ER and AR are composed of several functional domains, such as the N-terminal domain (NTD), the DNA-binding domain (DBD), and the ligand-binding domain (LBD) (McEwan 2009). The investigation of PDB structures show that except the native hormone-binding pocket, there are activation function 2 (AF-2) pocket and binding function 3 (BF-3) pocket located on the ligand-binding domain (LBD) of the ER and AR (Estébanez-Perpiñá et al., 2007; Axerio-Cilies et al., 2011; Lack et al., 2011; Buzón et al., 2012). Compounds bound to either of the pockets are demonstrated to interfere with the protein functions and the related signal pathway as agonists or antagonists (Moore et al., 2010; Nwachukwu et al., 2017) (Figure 1A). Even for the same pocket, such as the native ligand-binding pocket, considerable conformational changes occurred upon various types of compound binding (Min et al., 2021) (Figure 1B).

Current used small molecule docking programs such as AutoDock Vina can only consider the flexibility of the small molecule while keeping the protein conformation fixed (Trott and Olson 2010). The bias will be introduced only when one of the protein structures is used as the receptor for docking experiments. To integrate the conformation change information from the resolved structures, here we built docking models based on all available complex structures of the ER and AR and constructed a docking server (Estrogen and Androgen Receptor Database, EARDB, http://eardb.schanglab.org.cn/). The user can easily dock the compound of interest to the conformational ensembles of ERα, ERβ, and AR by several simple clicks. The top 10 highest docking score poses among all the ensembles are returned. The compound fit to any of the pocket may be potential EDCs of the ER or AR. In addition, the 2D similarity search function for the known ER or AR modulators is also implemented based on data retrieved from the binding database. The aim is to provide a tool to predict any possible ER or AR effectors with multi-conformational docking models and ligand-based similarity.

## Material and Methods

### Construction of Docking Models

By using the advance search option, the structures of ERα, ERβ, and AR are retrieved from the PDB (the Protein Data Bank) with the uniprot accession numbers of P03372, Q92731, and P10275. The structures with small molecules bound with the protein are used to generate the docking models. Totally, 282, 29, and 81 PDB structures are downloaded for proteins ERα, ERβ, and AR, respectively. For a crystal structure with multiple chains, each chain is separated and treated as a unique docking model. Then, the protein structure and ligand structure are split into different files. AutoDock Vina is chosen as the docking tool due to its excellent performance in systematic docking program evaluations (Trott and Olson 2010; Wang et al., 2016). The protein structures are operated by Receptor_prepare.py to remove waters and add polar hydrogens and Kollman charges to obtain the receptor pdbqt files. The docking box is defined by using the geometric center of the native binding ligand from the original PDB as the box center with 28 × 28 × 28 Å in size to include the entire binding site. Ligand_prepare.py is used to generate the ligand pdbqt file. The flexible bonds are set as default, and the Gasteiger charge is computed for the ligand. Both the receptor and ligand pdbqt files are generated in the neutral pH condition. The top ten docking poses are allowed to output with the docking score. In the re-dock experiment, the native binding ligands are docked into the corresponding receptors to validate the docking protocol. The receptor files which possess docking poses less than 2 Å rmsd with the native binding ligand are incorporated to the web server to evaluate the user-submitted small molecules.

### Activity Data Curation

We also collected the activity data of the reported ERα, ERβ, and AR agonist or antagonist from the binding database (http://www.bindingdb.org) (Chen et al., 2001; Liu et al., 2007; Gilson et al., 2016) to enable the user to evaluate the 2D similarity of his own compound and the known ER/AR modulators. All the records with the uniprot accession numbers of P03372, Q92731, and P10275 are retrieved from the binding database and implemented in the local server.

### Structure Similarity Search

In the structure similarity search function, the smi, mol2, or sdf file of the interested compound need to be provided by the user. The Tanimoto similarity coefficient between the user-uploaded small molecule and the ligands in the EARDB is computed by Open Babel 2.3.2 based on the linear fingerprint of fragment indices. The Tanimoto coefficient is a value range from 0 to 1, representing the level of similarity between two molecules. The value of 1 is the highest similarity and indicates the same molecules, and the value of 0 is the lowest similarity. The EARDB will automatically calculate the Tanimoto value for each ligand in the database with the query compound and will only return the ligands with similarity values higher than the user-defined cutoff with a descending rank.

### Implementation

The EARDB is installed on CentOS 7.6 server workstations. The webserver platform is constructed by Apache 2.4.6, and the website was built with PHP 5.6.4. The MySQL 5.7 Database management system was used to organize, manage, and sort data of various types. The open-source Java viewer NGL is embedded on the webpage for 3D molecular visualization (https://nglviewer.org/). Open Babel 2.3.2 is used for format transformation, 3D coordinate generation, and 2D similarity search for the uploaded files (O'Boyle et al., 2011). AutoDock Vina 1.1.2 (Trott and Olson 2010) is used to obtain the docking scores and binding modes with default settings.

## Results and Discussion

### Overview of the Database

**FIGURE 2:**
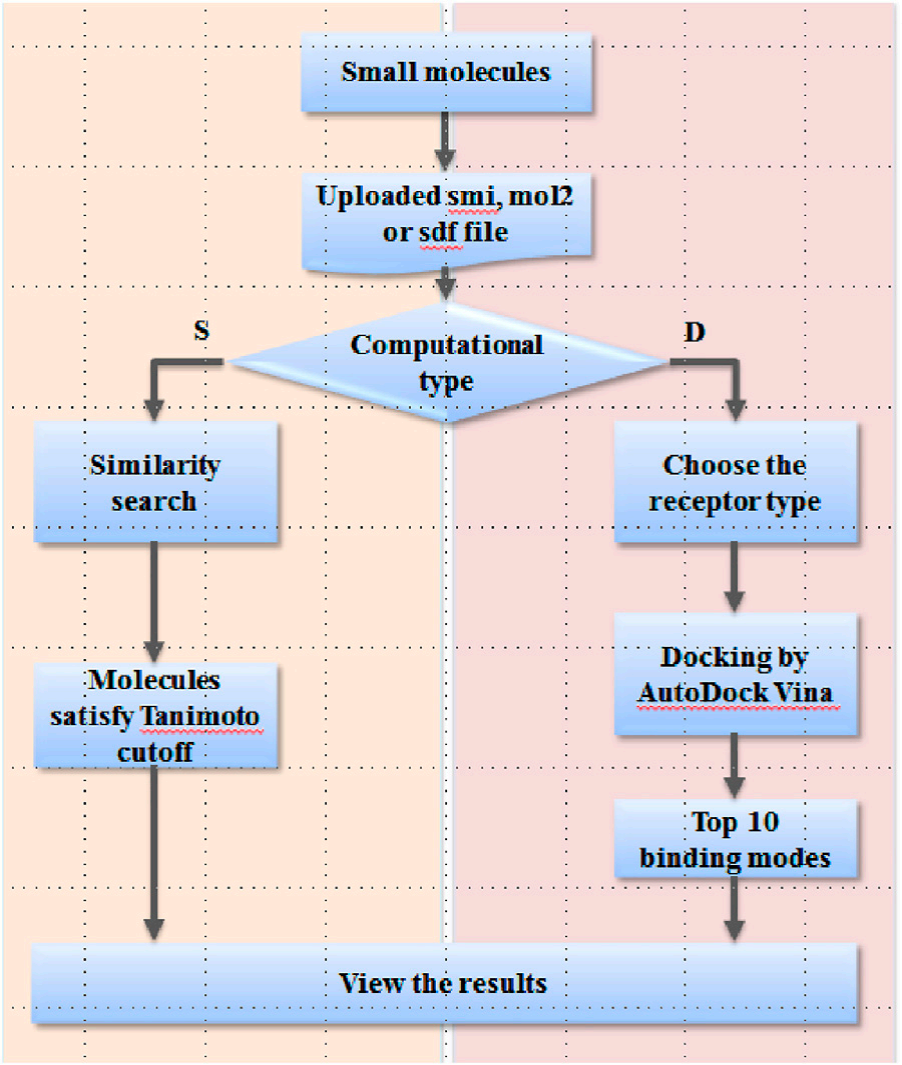
Workflow of the EARDB.

The EARDB (http://eardb.schanglab.org.cn/) currently implements two major functions, the 2D chemical similarity search for known ER/AR modulators and the online docking module to predict the potential ER/AR modulator (Figure 2). The ligand database contains about 7,800 unique compounds associated with 13,190 related activity records of the ER and AR from *Homo sapiens*. For each ER/AR modulator entry, the molecular chemical name, the 2D chemical structure and monomer ID from the binding database are provided. The online docking module provides a web-based interface to predict the binding mode and binding affinity for the user-uploaded compounds with the protein of interest. There are three types of protein targets, including ERα, ERβ, and AR. For each type of target, structures in complex with differential compounds are retrieved from the RCSB Protein Data Bank (Rose et al., 2011). Totally, 580, 62, and 91 docking models derived from the experimental structures of ERα, ERβ, and AR are available on the server.

The workflow of the web server is shown in Figure 2. For the user-interested compound, the strict smi, mol2, or sdf format file is needed to upload to the server. Two computational types are provided as following: “S” for similarity search and “D” for docking. For similarity search, the Tanimoto cutoff needs to be defined by the user. By submission of the job, Open Babel 2.3.2 is launched on the server to retrieve any compounds with Tanimoto values greater than the cutoff value. A molecular table is presented on the webpage to display the results, and also, a tab-delimited txt file is provided to download. For the docking module, the target type needs to be selected as the first step. By submission of the job, ligand preparation, a series of molecular docking experiments against all structures of the specific target type will be automatically carried out on the EARDB server. The top 10 models ranked by the predicted binding affinities are kept and visualized in 3D by NGL. A package of docking results is also provided to download from the results page.

### Validation of Docking Models

**FIGURE 3:**
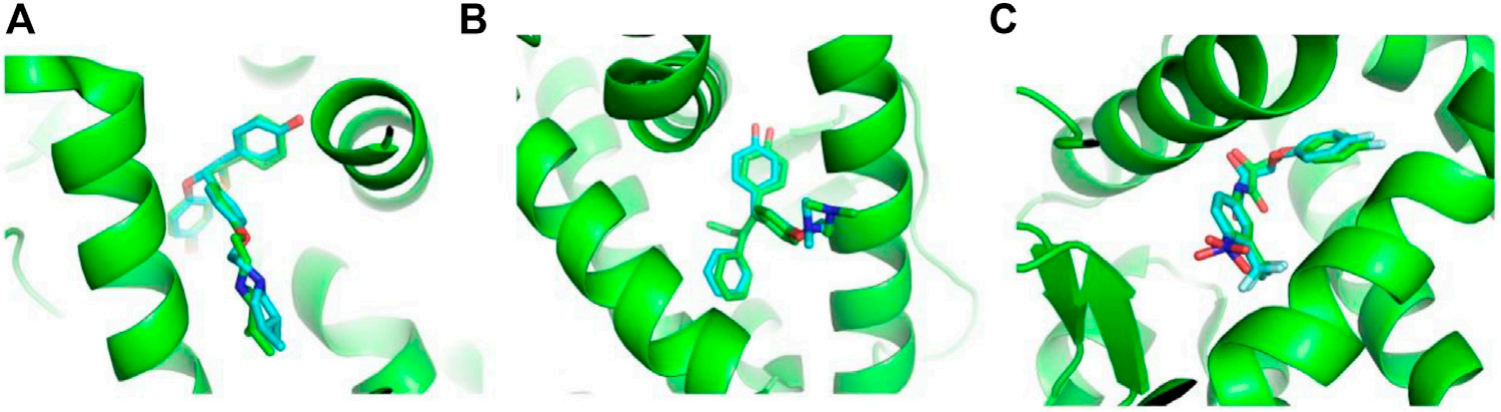
Representative poses from re-dock experiments of ERα **(A)**, ERβ, and **(B)** AR **(C)**. The proteins are represented in a green cartoon model, and ligands are represented in a stick model. The carbon atoms from crystal structures are shown in green color and those from the docking pose are shown in cyan.

To consider the conformational change of proteins upon binding different ligands, we retrieved all the protein–small molecule complex structures of ERα, ERβ, and AR from the PDB website. As shown in Table 1, there are 282, 32, and 81 complex structures for ERα, ERβ, and AR, respectively. Based on these structures, totally 621, 66, and 105 docking models were obtained by separating chains. Then, the re-dock experiments were performed to dock the native ligand from the experimental structures to the protein active site. It is necessary to validate the parameters set in the docking protocol, as well as if the protein structure is qualified for docking. The root mean square deviation (RMSD) between the docking poses generated by Vina and the native ligand structure from the original experimental structure are used to evaluate the accuracy of re-dock experiments. As shown in Table 1 and Supplementary Table S1, among 780 docking models, 733 models obtained docking poses with RMSD less than 2.0 Å (Supplementary Table S1). The re-dock results of 1XP1 (ERα) (Blizzard et al., 2005), 2FSZ (ERβ) (Wang et al., 2006), and 2AX8 (AR) (Bohl et al., 2005) are shown here as examples. The docking poses and the original crystal structures are presented in Figure 3. The RMSD values of 1XP1 (ERα), 2FSZ (ERβ), and 2AX8 (AR) are 1.48, 0.87, and 1.12 Å, respectively. The docking poses superimpose well with the native ligand structure from the original crystal structures, indicating the docking procedure is able to recover the experimental structures. To include more conformational diverse structures, the docking models with RMSD less than 2.0 Å are considered as successful docking systems and kept in the receptor database to provide readily to dock function on the web server.

**TABLE 1:** Number of structures and docking models for ERα, ERβ, and AR.

Protein name	Number of crystal structures with the small molecule ligand	Number of docking modes in re-dock experiment	Number of successful docked systems in re-dock experiment^a^
ERα	282	609	580
ERβ	32	66	62
AR	81	105	91

a Docking models with the lowest RMSD of docking poses less than 3.0 Å are defined as successful docking systems.

### Multiple Conformation–Based Docking

**FIGURE 4:**
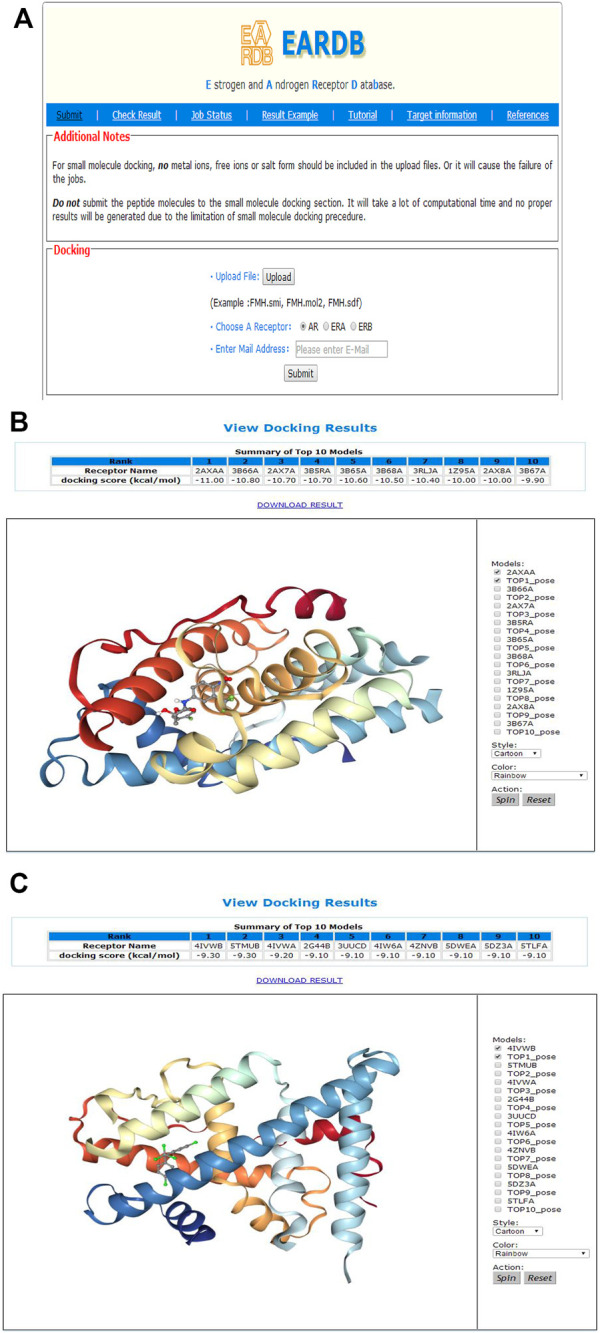
**(A)** Input webpage for multi-conformational docking of ERα, ERβ, or AR; **(B)** The result page of multi-conformational docking (example running for FHM); **(C)** The result page of multi-conformational docking (example running for DDT).

Multiple structures of ERα, ERβ, and AR are collected from the PDB database and prepared as docking models. The user could upload the smi, sdf, or mol2 file of one small molecule and then choose a receptor for docking (Figure 4A). An email address is needed to receive the message of job submission and job status. After the job is completed, a notification email will be sent to the user’s email address. An investigation drug of AR, ligand name from the PDB as FHM, was used as an example for multiple conformational docking. FHM is a native ligand in the crystal structure with PDBID as 2AXA. The sdf file of FHM was uploaded, and the receptor type of the AR was selected. With one click on submission button, the job is submitted to the server conveniently. After about 3 h running, ninety-three docking experiments were finished. On the “Job Status” page, it is shown that the job was completed. By click the result link from the email or by search for the job ID in the “Check Result” page, the result page can be accessed. As shown in Figure 4B, a table of the top 10 lowest energy poses is provided in the result webpage. The PDBID of the receptor model and the corresponding docking score are presented. For FHM, its original receptor file 2AXA (chain A) obtained the lowest docking score as −11.0 kcal/mol. The binding poses could also be explored by the NGL molecule viewing window on the page. As the docking score of Vina is fitted to the binding affinity, molecules with a low docking score may be a possible modulator.

DDT (4,4-dichlorodiphenyltrichloroethane) is used as another example. As a pesticide, DDT was banned because it acts as an endocrine-disrupting chemical with ERα agonist activity (Nwachukwu et al., 2017). The sdf file of the compound was uploaded to the server, and ERα was chosen as the receptor to perform the multi-conformation–based docking. The results are displayed in Figure 4C. It is showed that the compound bound well with various PDB structures of ERα, and the top ten lowest docking scores ranged from −9.3 to −9.1 kcal/mol, indicating DDT as a strong ERα binder (Figure 4C).

### 3.4 2D Similarity Search for Known ERα, ERβ, or AR Modulators

**FIGURE 5:**
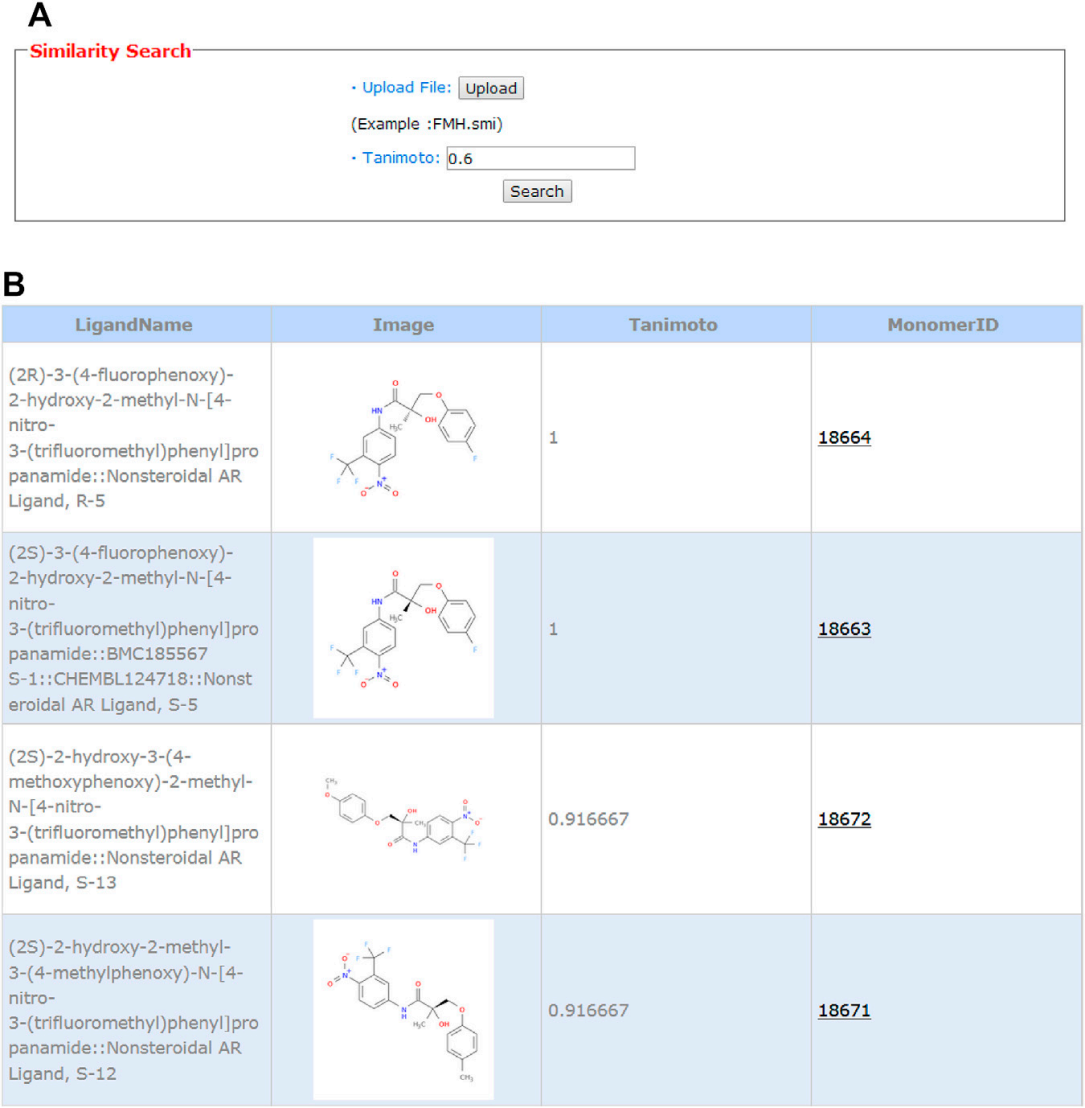
Input **(A)** and output **(B)** webpages for 2D similarity search.

Ligand-based chemical structure similarity search is provided on the web server. As shown in Figure 5A, the user can choose a molecule in the smiles format from the local storage and upload it to the server. Here, we also take FHM as an example. The default value of 0.6 is taken as the Tanimoto cutoff. As shown in Figure 5B, the monomerID from the binding database, ligand name, 2D chemical structure, and Tanimoto value are presented. A hyperlink is added to the monomer ID, and by clicking the ID, the user will be led to the binding database webpage for detailed information, such as activity values, assay description, and publication. The first hit in the table with the Tanimoto value of 1.0 is the compound itself. Molecules highly similar to the potent modulator may have the same function and could be an endocrine active compound.

## Conclusion

A web server, EARDB database, was constructed to predict the potential ERα, ERβ, and AR modulators. Both structure-based methods, the multi-conformation docking, and ligand-based method, and 2D similarity search are provided on the server. The investigation of the available PDB structures of ERα, ERβ, and AR showed that there are several ligand binding sites on these targets and considerable binding site plasticity. Thus, over 600 docking models are prepared and allowed to settle on the web server to provide docking against the conformational ensemble of each target type. The results of re-dock experiments suggest the docking procedures could reproduce most of the experiment structures of the protein–small molecule complexes. The server provides either the protein structure–based docking function or ligand-based similarity search function to estimate if the query compound is a potential ERα, ERβ, or AR modulator. It will benefit for either the ER or AR drug design or EDC prediction.

## Data Availability

The original contributions presented in the study are included in the article/Supplementary Material, further inquiries can be directed to the corresponding authors.
